# Very Late Fracture of a First-Generation Drug-Eluting Stent

**DOI:** 10.1016/j.jaccas.2026.107396

**Published:** 2026-03-14

**Authors:** Flora Tsakirian, Dimitrios Afendoulis, Sotirios Tsalamandris, Andreas Synetos, Konstantinos Tsioufis, Konstantinos Toutouzas

**Affiliations:** a Unit of Structural and Valvular Heart Diseases, First Department of Cardiology, National and Kapodestrian University of Athens, Hippokration General Hospital of Athens, Athens, Greece; b European University Cyprus, Engomi, Cyprus

**Keywords:** drug-eluting stent, in-stent restenosis, percutaneous coronary intervention, stent fracture

## Abstract

**Background:**

Coronary stent fracture, most frequently associated with first-generation drug-eluting stents, is an uncommon yet clinically relevant cause of very late stent failure.

**Case Summary:**

A 67-year-old man with dyslipidemia, smoking history, hypertension, and prior percutaneous coronary intervention 15 years ago underwent coronary angiography as part of the preoperative evaluation for cholecystectomy. Angiography revealed fracture of a first-generation sirolimus-eluting stent (3.0 × 26 mm) with displacement of struts in the mid left anterior descending artery, with preserved TIMI flow grade III. He presented no symptoms suggestive of angina, and ischemia evaluation with stress echocardiography was negative. A conservative management strategy was adopted.

**Discussion:**

This case highlights the long-term mechanical limitations of early-generation drug-eluting stents and underlines the importance of individualized decision-making guided by symptoms and objective ischemia assessment.

**Take-Home Messages:**

Very late stent fracture may occur decades after implantation, particularly with the use of first-generation drug-eluting stents. Management should be guided by patient symptoms and evidence of ischemia rather than angiographic appearance alone.

## Visual Summary

**Figure:**
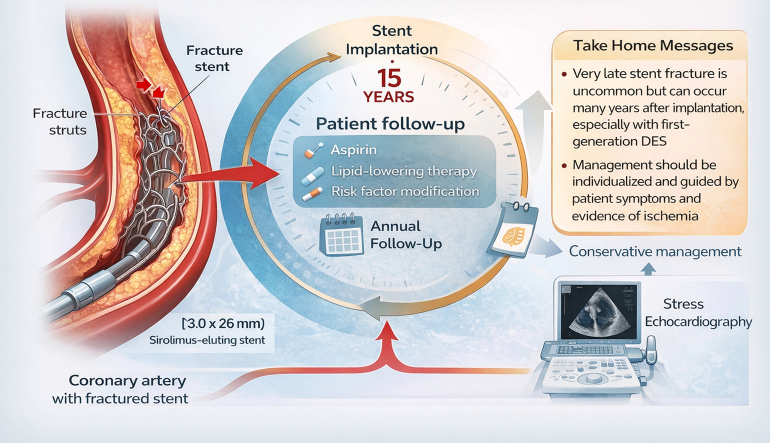

## History of Presentation

A 67-year-old man with dyslipidemia, smoking, hypertension, and prior percutaneous coronary intervention (PCI) 15 years ago owing to a non–ST-segment elevation myocardial infarction acute coronary syndrome underwent coronary angiography as part of the preoperative evaluation for cholecystectomy. His medical therapy consisted of aspirin 100 mg, rosuvastatin 40 mg, amlodipine 5 mg, and olmesartan 40 mg. The patient had reported mild dyspnea on exertion, and the heart team decided to perform coronary angiography to further investigate the patient preoperatively given his cardiovascular history. He did not report chest pain or syncope. Physical examination was unremarkable, with normal vital signs and no signs of heart failure.

## Differential Diagnosis

Considerations included in-stent restenosis, stent thrombosis, coronary artery aneurysm, and mechanical stent failure (fracture or deformation).

## Investigations

**Figure 1:**
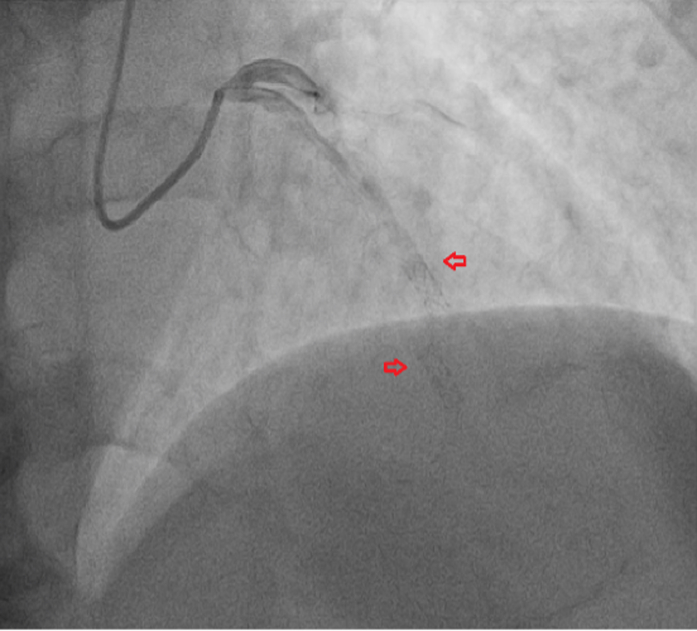
Coronary Angiography Showing the Fracture of First-Generation Drug-Eluting Stent (Arrows) With Displacement of the Struts

**Figure 2:**
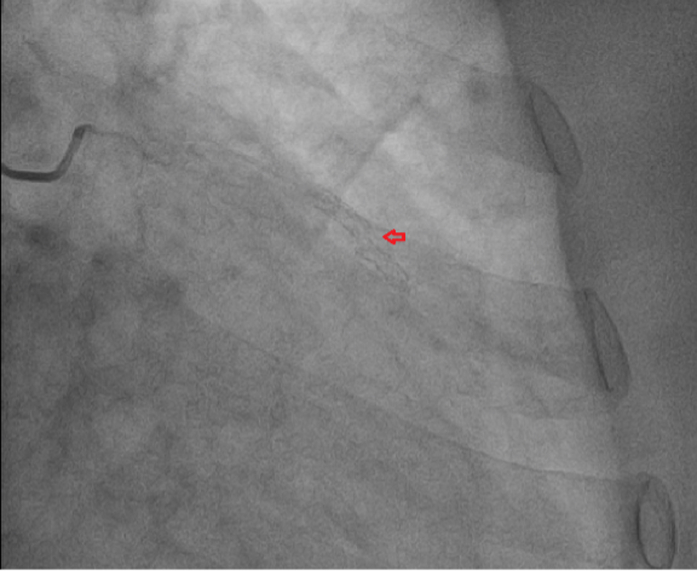
Coronary Angiography Showing the Fracture of First-Generation Drug-Eluting Stent (Arrow)

**Figure 3:**
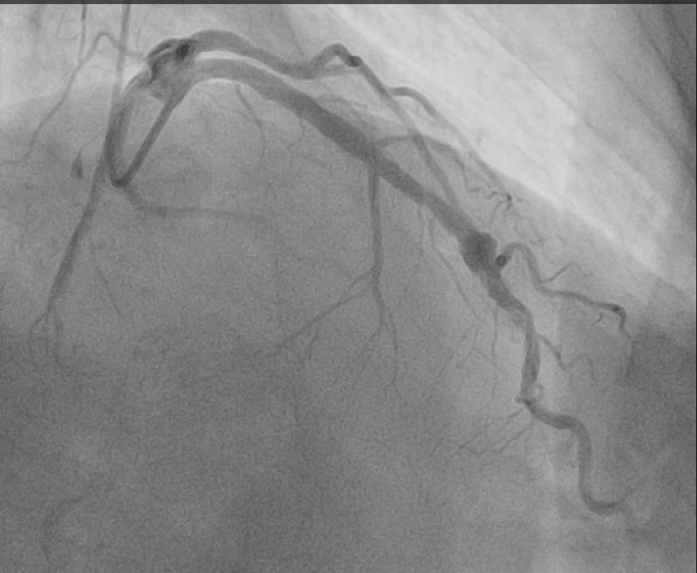
Coronary Angiography Showing the Flow in the Vessel With the Fracture of First-Generation Drug-Eluting Stent

Coronary angiography demonstrated fracture of the previously implanted first-generation sirolimus-eluting stent (3.0 × 26 mm) with displacement of struts that were implanted in the mid left anterior descending artery (Figures 1 and 2). Despite the mechanical disruption, antegrade coronary flow was preserved (TIMI flow grade III) (Figure 3), and no significant luminal compromise was observed.

Given the angiographic findings, functional assessment was followed by stress echocardiography. The echocardiographic study showed no inducible ischemia in the left anterior descending artery territory.

## Management

In the absence of symptoms or objective evidence of myocardial ischemia, a conservative management strategy was chosen. Ezetimibe was added to the patient's daily medical regimen to lower his low-density lipoprotein cholesterol to <55 mg/dL, as proposed by the 2024 European Society of Cardiology (ESC) guidelines on chronic coronary syndromes.^1^ He remained on a single-antiplatelet regimen with aspirin given the fact that no revascularization was carried out and there was no troponin elevation or acute coronary syndrome. Emphasis was placed on risk-factor modification and more specifically, smoking cessation. The 2024 ESC guidelines on chronic coronary syndromes also support repeat revascularization with PCI or coronary artery bypass grafting in patients with stent fracture who are symptomatic or have demonstrable ischemia.^1^ As these criteria were not met, invasive reintervention was deferred.

## Outcome and Follow-Up

The patient remained clinically stable, asymptomatic without development of angina or other ischemic symptoms during follow-up. No adverse cardiovascular events were reported. Continued clinical surveillance was planned, with cardiology follow-up at 1 year or earlier in the presence of symptoms. Cholecystectomy was carried out successfully and without complications under continuation of aspirin, as proposed by the 2022 ESC guidelines for noncardiac surgery.^2^

## Discussion

Coronary stent fracture is a rare but important complication, increasingly recognized during long-term follow-up PCI. It was first described predominantly with first-generation drug-eluting stents (DESs) such as the sirolimus-eluting stent, which were characterized by thicker struts and lower flexibility compared with contemporary devices.

Predisposing factors for stent fracture include vessel tortuosity, heavy calcification, long stent length, overlapping stents, hinge motion, and location in dynamically mobile coronary segments. The left anterior descending artery, particularly in its midsegment, may be exposed to repetitive mechanical stress over time. Clinical consequences of stent fracture vary widely, ranging from incidental findings with preserved flow to restenosis, aneurysm formation, stent thrombosis, acute coronary syndromes, and sudden cardiac death. Importantly, angiographic appearance alone does not guide revascularization. Current practice emphasizes symptom assessment and objective ischemia testing to guide management decisions.^3^

This case is noteworthy given the exceptionally late presentation 15 years after implantation, as well as the absence of ischemia despite clear mechanical failure. This complication was discovered in the context of preoperative assessment of a patient with established cardiovascular disease who needed to undergo intermediate- to high-risk noncardiac surgery. As proposed by the 2022 ESC guidelines for noncardiac surgery, except for functional assessment and evaluation of electrocardiogram and biomarkers, these patients should undergo further diagnostic evaluation after cardiology consultation prior to surgery.^2^

This case reinforces the importance of individualized patient management and illustrates the long-term mechanical limitations of early-generation stent platforms, while highlighting the improved durability of modern DESs. Moreover, the importance of uninterrupted single-antiplatelet treatment during noncardiac surgery is highlighted in our case.

**Visual Summary:**
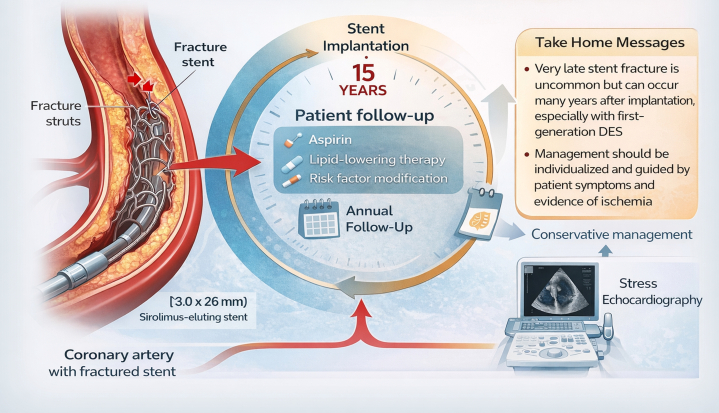
Main Learning Points Regarding Coronary Stent Fracture DES = drug-eluting stent.

## Conclusions

Very late fracture of first-generation DESs can occur decades after implantation and may be discovered incidentally. In asymptomatic patients with preserved coronary flow and no evidence of ischemia, conservative management aligned with guideline-recommended practice is appropriate.

## Funding Support and Author Disclosures

The authors have reported that they have no relationships relevant to the contents of this paper to disclose.

**Take-Home Messages**

• Very late mechanical failure remains a limitation of first-generation drug-eluting stents and may present incidentally years after implantation.
• Management of stent fracture should be guided by symptoms and objective ischemia assessment rather than angiographic findings alone.

## Appendix

Video 1 Coronary Angiography Left Anterior Oblique View Showing the Presence of a Stent Fracture With Struts Displacement and TIMI Flow Grade III
